# Complete Genome Sequence of the Fish Pathogen *Flavobacterium columnare* Pf1

**DOI:** 10.1128/genomeA.00900-16

**Published:** 2016-09-01

**Authors:** Yulei Zhang, Pin Nie, Li Lin

**Affiliations:** a Department of Aquatic Animal Medicine, College of Fisheries, Huazhong Agricultural University, Wuhan, China; b State Key Laboratory of Freshwater Ecology and Biotechnology, Institute of Hydrobiology, Chinese Academy of Sciences, Wuhan, China

## Abstract

*Flavobacterium columnare* is the etiologic agent of columnaris disease, a devastating fish disease prevailing in worldwide aquaculture industry. Here, we describe the complete genome of *F. columnare* strain Pf1, a highly virulent strain isolated from yellow catfish (*Pelteobagrus fulvidraco*) in China.

## GENOME ANNOUNCEMENT

*Flavobacterium columnare* belongs to the family *Flavobacteriaceae* and is the causative agent of columnaris disease, which causes substantial mortality in numerous freshwater fish worldwide (1–4). There are three genomovars, in accordance with differences in 16S rRNA sequences and restriction fragment length polymorphism (RFLP) (5–9). *F. columnare* strain Pf1 was isolated from the diseased yellow catfish (*Pelteobagrus fulvidraco*) in China (10). It belongs to genomovar I and is also virulent in other species of freshwater fish, such as the mandarin fish (*Siniperca chuatsi*) (10). Built on single-molecule real-time (SMRT) technology, the scalable high-throughput Sequel system delivers unprecedented sequencing results through long reads, uniform coverage, and high consensus accuracy (11), and we used it to sequence and analyze the full genome of the Pf1 strain.

*F. columnare* Pf1 was cultured on Shieh medium at 28°C. Genomic DNA was isolated using the TIANamp bacteria DNA kit (Tiangen Biotech, Beijing, China). The stock DNA was separated into three aliquots, one for SMRT sequencing, one for Illumina sequencing, and the other for gap closing. The SMRT sequencing work was conducted using PacBio RSII system. A total of 266,951 cleaned subreads totaling 663,416,366 bases were obtained, resulting in a 210-fold coverage of the genome. Assembly was performed using the HGAP software and produced 12 contigs with total contig lengths of 3,233,416 bp. The next-generation sequencing by HiSeq 2000 (Illumina) was performed according to the manufacturer’s instructions (Shanghai Oebiotech Co., Ltd., Shanghai, China). A total of 4,617,419 paired reads were obtained. The Illumina data then were mapping on previous assembled contigs using Bowtie2 software. After removal of redundant sequences, we obtained three contigs with a total length of 3,165,083 bp. Three gaps were then filled by sequencing the PCR products. The DNA sequence was submitted to the NCBI’s Prokaryotic Genome Automatic Annotation Pipeline (PGAAP) for annotation, and then the annotated genome was submitted to GenBank.

The complete genome of *F. columnare* consists of 3,171,081 bp, with an average G+C content of 31.58%. The genome is 86.19% coding and has 2,883 predicted genes consisting of 2,784 protein-coding, 18 rRNA, and 81 tRNA genes. For the protein-coding genes, the average length is 982 bp, and 1,664 genes (59.77%) have assigned functions. Analysis of the *F. columnare* genome sequences reveals many features common to other sequenced *Flavobacterium* species. As expected, outer membrane protein clusters, such as ABC transports, OmpA and OmpH family outer membrane proteins, TonB-dependent outer membrane receptors, and precursors were identified in this strain, which are important surface structures of bacteria in identification, adhesion, and infection. Meanwhile, we identified some well-known virulence factors, such as hemolysin, adhesin SprB, and SprC. These factors are the symbols of strong virulent strain and might be closely related with its pathogenesis in fish. Genomic analysis is the important step in understanding the pathogenesis of *F. columnare* and will facilitate the development of an effective vaccine for the prevention of columnaris disease.

### Accession number(s).

The genome sequence was deposited in GenBank under accession no. CP016277.

## References

[1] Davis HS 1922 A new bacterial disease of fresh water fishes. US Bur Fish Bull 38:261–280.

[2] Pacha RE, Ordal EJ 1970 Myxobacterial diseases of salmonids, p 243–257. *In* Snieszko SF (ed), A symposium on diseases of fishes and shellfishes. American Fisheries Society, Washington, DC, Special Publication.

[3] Austin B, Austin DA 1999 Bacterial fish pathogens: disease of farmed and wild fish. Heriot-Watt University, Edinburgh, Scotland, United Kingdom.

[4] Decostere A, Haesebrouck F, Devriese LA 1998 Characterization of four *Flavobacterium columnare* (*Flexibacter columnaris*) strains isolated from tropical fish. Vet Microbiol 62:35–45. doi:10.1016/S0378-1135(98)00196-5. 9659690

[5] Bernardet JF, Segers P, Vancanneyt M, Berthe M, Kersters K, Vandamme P 1996 Cutting a Gordian knot: emended classification and description of the genus *Flavobacterium*, emended description of the family *Flavobacteriaceae*, and proposal of *Flavobacterium hydatis* nom. nov. (basonym, *Cytophaga aquatilis* Strohl and Tait 1978). Int J Syst Evol Bacteriol 46:128–148.

[6] Triyanto A, Wakabayashi H 1999 Genotypic diversity of strains of *Flavobacterium columnare* from diseased fishes. Fish Pathol 34:65–71. doi:10.3147/jsfp.34.65.

[7] Arias CR, Welker TL, Shoemaker CA, Abernathy JW, Klesius PH 2004 Genetic fingerprinting of *Flavobacterium columnare* isolates from cultured fish. J Appl Microbiol 97:421–428. doi:10.1111/j.1365-2672.2004.02314.x. 15239710

[8] Darwish AM, Ismaiel AA 2005 Genetic diversity of *Flavobacterium columnare* examined by restriction fragment length polymorphism and sequencing of the 16S ribosomal RNA gene and the 16S−23S rDNA spacer. Mol Cell Probes 19:267–274. doi:10.1016/j.mcp.2005.04.003. 15979275

[9] Shoemaker CA, Olivares-Fuster O, Arias CR, Klesius PH 2008 *Flavobacterium columnare* genomovar influences mortality in channel catfish (*Ictalurus punctatus*). Vet Microbiol 127:353–359. doi:10.1016/j.vetmic.2007.09.003. 17964085

[10] Zhang YL, Zhao LJ, Zhou WD, Ai TS, Lin L 2016 Characterization and pathogenicity of a *Flavobacterium columnare* isolated from *Pelteobagrus fulvidraco*. J Huazhong Agricul Univ 35:27–33. (In Chinese.)

[11] Gupta PK 2008 Single-molecule DNA sequencing technologies for future genomics research. Trends Biotechnol 26:602–611. doi:10.1016/j.tibtech.2008.07.003. 18722683

